# Comparing Light—Emitting—Diodes Light Traps for Catching *Anopheles* Mosquitoes in a Forest Setting, Western Thailand

**DOI:** 10.3390/insects12121076

**Published:** 2021-11-30

**Authors:** Pairpailin Jhaiaun, Amonrat Panthawong, Manop Saeung, Anchana Sumarnrote, Monthathip Kongmee, Ratchadawan Ngoen-Klan, Theeraphap Chareonviriyaphap

**Affiliations:** 1 Department of Entomology, Faculty of Agriculture, Kasetsart University, Bangkok 10900, Thailand; pairpailin.j@hotmail.com (P.J.); aor_bio@hotmail.com (A.P.); manop.saeu@ku.th (M.S.); fagrrwn@ku.ac.th (R.N.-K.); 2 Department of Entomology, Faculty of Agriculture at Kamphaeng Saen, Kasetsart University, Kamphaeng Saen Campus, Nakhon Pathom 73140, Thailand; fagraas@ku.ac.th (A.S.); fagrmtk@ku.ac.th (M.K.)

**Keywords:** malaria, vector, light traps, mosquitoes, UV fluorescent, wavelength

## Abstract

**Simple Summary:**

A field study was conducted in a forest to compare the effectiveness of light traps fitted with different bulbs across the wavelength spectrum. Ultraviolet (UV) fluorescent light was found to be most effective to collect adult *Anopheles* mosquitoes from 21:00 h to the pre-dawn hours in the dry season. These findings have important implications for monitoring vector density in the endemic malaria areas where other methods cannot be executed. A more comprehensive and systematic study of how mosquitoes respond to light would benefit Thailand’s national control program. Their potential for more precisely sampling vectors holds promise as a tool for mosquito monitoring endemic malaria areas and outbreak hotspots.

**Abstract:**

Light traps are a common method for attracting and collecting arthropods, including disease vectors such as mosquitoes. Various types of traps have been used to monitor mosquitoes in a forest in Western Thailand. In this study, four Light Emitting Diodes (LED) light sources (UV, blue, green, and red) and two fluorescent lights (white and UV) were used to trap nocturnal adult mosquitoes. These traps were used with light alone and not any additional attractant. The experiment was conducted from 18:00 to 06:00 h. on six consecutive nights, every two months, across dry, wet, and cold seasons. All specimens were first identified by morphological features and subsequently confirmed by using PCR. We collected a total of 873 specimens of 31 species in four genera, *Anopheles*, *Aedes*, *Culex*, and *Armigeres*. *Anopheles harrisoni* was the predominant species, followed by *Aedes* *albopictus*, *Culex* *brevipalpis*, *Culex nitropunctatus*, and *Armigeres* (*Leicesteria*) *longipalpis*. UV fluorescent light was the most effective light source for capturing forest mosquitoes, followed by UV LED, blue LED, green LED, white fluorescent, and red LED. The optimal times for collection were from 21:00 to 03:00 h in the dry season. Our results demonstrate that appropriate sampling times and light sources should be selected for optimal efficiency in vector surveillance programs.

## 1. Introduction

Mosquitoes are well-recognized as important arthropod vectors that are responsible for transmitting many medically important pathogens and parasites, including viruses, bacteria, protozoans, and nematodes, which cause serious diseases, such as malaria, dengue, chikungunya, encephalitis, and filariasis [1]. Of these, malaria is a serious and sometimes fatal disease caused by a parasite transmitted to humans via *Anopheles* mosquitoes. Interventions can be implemented to mitigate and reduce the risk of infection and prevent disease. Long-lasting insecticidal nets and indoor residual spraying have long been used as the main interventions to combat malaria indoors [2]. Despite the efficacy of these methods, malaria cases still occur when vector mosquitoes evade control measures by feeding outdoors. Thus, controlling outdoor transmission represents a significant challenge, especially in Southeast Asia [3]. Mosquito sampling and monitoring are essential for developing disease control programs, and improving surveillance techniques will contribute to accelerating research efforts for preventing outdoor transmission.

Several approaches can be used to understand vector density and dynamics, including larval collection, light traps, and indoor resting collection [4,5]. Some techniques use humans or live animals as bait to attract adult female mosquitoes [6,7]. The cow-baited net trap, which involves placing a single adult cow inside an enclosure made of bed net, has been used to collect host-seeking mosquitoes that rest on the net after engorging a cow’s blood [8]. However, this may raise an ethical issue in some places, such as India, where cows are idolized. Hence, because these approaches pose a risk of infection during mosquito collection, alternative methods are needed for mosquito surveillance and monitoring. There are some traps that have been utilized together with synthetic compounds and other chemical cues to lure female adult mosquitoes [9,10]. Light traps are frequently used to catch large numbers of mosquitoes for laboratory studies, such as for virus isolation [11,12]. The type of light trap selected in mosquito studies is typically based on the need to catch as many mosquitoes of the desired species as possible [13]. Light trapping methods have been used for a many decades to monitor populations of mosquito vectors without live bait cues [14]. They are a relatively safe approach that circumvents the need for human contact with mosquitoes [15]. They also eliminate the often lengthy and burdensome requirement for ethical approval. Light-emitting diodes (LEDs) are often used in light traps because they are small and convenient to use, energy-efficient, and have superior battery longevity compared with incandescent bulbs [16]. Several studies have shown that LED lights can be used to attract various insects, including mosquitoes [15,17,18,19]. Many studies have used and assessed LEDs for mosquito trapping [10,20,21,22,23,24,25,26]. Mosquito responses to LED lights differ depending on the wavelengths of light emitted by a bulb; for example, green LED at 520 nm is a more attractive light source for *Anopheles* mosquitoes than blue LED at 470 nm [22]. However, information on attraction of LED light sources in *Anopheles* mosquitoes in Thailand is limited. In this study, we compared light traps equipped with fluorescent and LED light sources of different wavelengths for collecting adult mosquitoes in a forest setting.

## 2. Materials and Methods

### 2.1. Study Site

This study was conducted in the forest fringe area of Pu Teuy Village, Sai Yok District, Kanchanaburi Province (14°17′ N, 99°1′ E), approximately 250 km northwest of Bangkok. This area was selected based on a previous report that the region is inhabited by several species of *Anopheles* mosquitoes [27]. The region has a dry season (February to May), a wet season (May to October), and a cold season (November to February). Human activity is mostly related to agriculture, animal, farming, and forest activities.

### 2.2. Light Traps

**Figure 1:**
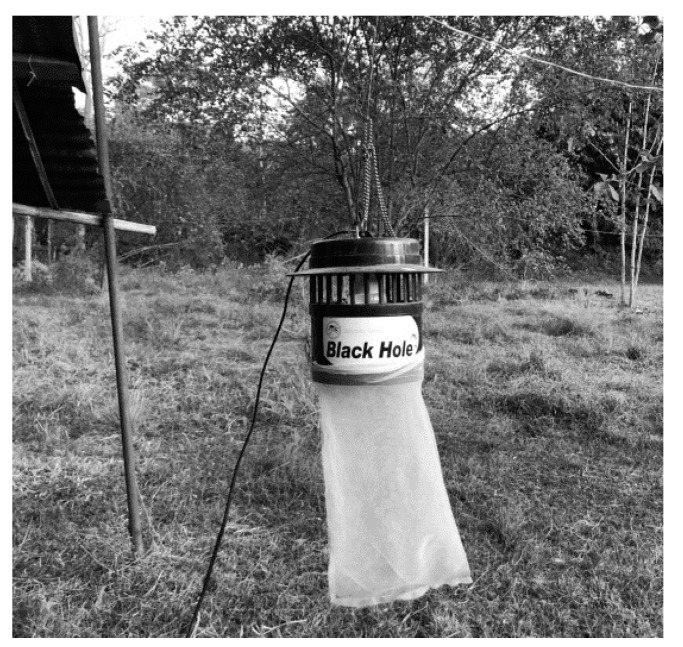
Mosquito traps (Black Hole™ Mosquito Trap, Bio-Trap Inc., Seoul, Korea) used in this study were purchased from Pan Science Co., Ltd., Bangkok, Thailand.

The mosquito traps (Black Hole™ Mosquito Trap, Bio-Trap Inc., Seoul, Korea) used in this study were purchased from Pan Science Co., Ltd., Bangkok, Thailand (Figure 1). The black plastic traps measure 25 × 25 × 32 cm and weigh 1.2 kg. The traps were equipped with an electrical fan [28] and an ultraviolet (UV) light source (two, 4-watt UV fluorescent bulbs) powered by an alternating current (AC) 220–240 V electrical system. The fluorescent light is the default light provided in the trap. Five traps were modified by replacing the factory-equipped light source with a 6-watt LED bulb representing the light spectrum (UV, blue, green, or red) or a 4-watt fluorescent bulb (white; Eve Lighting Co., Ltd., Bangkok, Thailand). A total of six traps were used in the experiment, with the following wavelength emission band ranges: UV fluorescent (354–468 nm; light intensity, 63,913 arbitrary units (a.u.)), white fluorescent (277–400 nm; light intensity, 53,791 a.u.), blue LED (416–428 nm; light intensity, 63,294 a.u.), green LED (553–567 nm; light intensity, 61,040 a.u.), UV LED (377–384 nm; light intensity, 63,554 a.u.), and red LED (740–755 nm; light intensity, 62,154 a.u.).

### 2.3. Mosquito Collection

Six trapping locations were established in the study area, approximately 200 m apart. Each light trap was hung approximately 150 cm above the ground level [29]. The experiment was conducted on six consecutive nights in each of the following months: February and April (dry season), June and August (wet season), and October and December (cold season), in 2020. During each of six consecutive trapping nights, the light traps were rotated among the six locations, using a Latin square design. Mosquito collection was conducted over 12 h, from 18:00 to 06:00 h. Each night, mosquitoes were collected from each trap every 3 h (at 21:00 24:00, 03:00, and 06:00 h). The collected mosquitoes were placed in a −20 °C freezer for 60 s and then morphologically identified.

### 2.4. Morphological Species Identification

Mosquitoes were separated from other insects, carefully examined by using a stereomicroscope, and identified according to sex. Species were morphologically identified based on the external features of proboscis, maxillary palpus, scutum, wing vein, spiracular-setae, legs, abdomen, and scales, using a standard taxonomic key [30,31,32,33,34,35]. All primary *Anopheles* species, including the *Anopheles minimus* complex, *Anopheles maculatus* group, and *Anopheles dirus* complex, were then stored at −20 °C for molecular identification.

### 2.5. DNA Extraction

All laboratory work was performed at the Department of Entomology, Faculty of Agriculture, Kasetsart University, Bangkok, Thailand. DNA from individual *An. minimus* complex, *An. maculatus* group, and *An. dirus* complex mosquitoes was extracted by using an EZNA^®^ tissue DNA kit (Omega Bio-Tek, Norcross, GA, USA). The final elution volume for DNA extractions was 50 μL. Distilled water was used as a negative extraction control. DNA solutions were stored at −20 °C until further use [36].

### 2.6. Molecular Species Identification

Multiplex allele-specific PCR assay (AS-PCR) was used to examine the internal transcribed spacer 2 (ITS2) genomic DNA region (Table 1). Members of the Minimus Complex (*An. aconitus*, *An. harrisoni*, *An. varuna*, and *An. minimus*) were identified following the procedure of [37] and members of the Maculatus Group (*An. maculatus*, *An. pseudowillmori*, *An. sawadwongporni*, and *An. dravidicus*) and Dirus Complex (*An. dirus*, *An. scanloni*, *An. cracens*, *An. baimaii*, and *An. nemophilous*) were identified by following the procedures of References [38,39], respectively.

### 2.7. Data Analysis

The numbers of each mosquito species captured by six different light traps were compared using the Kruskal–Wallis test by mean ranks. The efficacy of the traps to collect mosquitoes was evaluated by a generalized linear model (GLM). The total number of collected *Anopheles* mosquitoes per trap-night was treated as the response variable, and the light sources, seasons, and collection periods were defined as key factors. The goodness-of-fit model was validated by considering the deviance value; the optimal model was selected by mean deviance closest to one. The results from testing the model showed statistical significance for all tests with *p* < 0.05. To finalize, the parameter (key factor) that was a statistically significant predictor of the number of mosquitoes caught was used to determine the regression coefficients (B), standard errors, *p*-values, and 95% confidence intervals for the coefficients, using the Wald Chi-square test. Mosquito-trap efficacy was analyzed based on incidence rate ratio (IRR), which provided a standard incidence rate (IRR = 1) for comparison of variables. All data were analyzed by using the SPSS program (version 11.0; SPSS Inc., Chicago, IL, USA).

## 3. Results

A total of 873 adult mosquitoes were captured during the 36 trapping nights. More females (*n* = 818; 93.70%) than males (*n* = 55; 6.30%) were captured. The captured mosquitoes belonged to four genera: *Anopheles*, *Aedes*, *Culex*, and *Armigeres*. The most abundant genus was *Anopheles* (*n* = 514; 58.88%), followed by *Aedes* (*n* = 167; 19.13%), *Culex* (*n* = 148; 16.95%), and *Armigeres* (*n* = 44; 5.04%). The most abundant species in *Anopheles* was *An. harrisoni* (54.75%); in *Aedes*, it was *Ae. albopictus* (10.42%); in *Culex*, it was *Cx. brevipalpis* (8.36%); and in *Armigeres*, it was *Ar.* (*Lei*.) *longipalpis* (3.55%). Of the collected specimens, 4.24% could not be identified to the species level, due to damage.

Comparing among light sources, we found that the highest total number of mosquitoes was captured in the light trap fitted with UV fluorescent (*n* = 382; 43.76%), followed by UV LED (*n* = 177; 20.27%), blue LED (*n* = 133; 15.23%), green LED (*n* = 73; 8.36%), white fluorescent (*n* = 73; 8.36%), and red LED (*n* = 35; 4.01%). The highest number of individuals was captured by UV fluorescent for most genera, except *Armigeres*, for which the highest number was collected by using blue LED (Table 2).

For *Aedes* mosquitoes, the highest number of specimens was collected by using UV fluorescent light (*n* = 43; 25.75%), followed by blue LED (*n* = 40; 23.95%), UV LED (*n* = 23; 13.77%), green LED (*n* = 31; 18.56%), white fluorescent (*n* = 19; 11.38%), and red LED (*n* = 11; 6.59%; Table 2). The Kruskal–Wallis test by ranks showed that *Aedes* mosquitoes were non-significantly more responsive to UV fluorescent (mean rank, 122.92), followed by blue LED, UV LED, green LED, white fluorescent, and red LED (mean rank, 119.03, 110.89, 108.58, 103.39, and 83.19, respectively; *p* = 0.053; Table 3).

Of the *Anopheles* mosquitoes, 55.25% (*n* = 284) were caught by using the light trap with UV fluorescent, followed by UV LED (*n* = 115; 22.37%), blue LED (*n* = 54; 10.51%), white fluorescent (*n* = 34; 6.61%), green LED (*n* = 16; 3.11%), and red LED (*n* = 11; 2.14%; Table 2). The Kruskal–Wallis test by ranks showed that *Anopheles* mosquitoes were significantly more responsive to UV fluorescent and UV LED, with mean ranks of 144.56 and 127.82, respectively (Table 3). Significantly fewer specimens were collected from red LED, green LED, and white fluorescent, with the mean ranks of 92.69, 92.44, and 90.36, respectively (*p* < 0.001). The mean rank for blue LED was between the highest and lowest ranks at 103.13 (Table 3).

The highest number of *Armigeres* mosquitoes was collected by using blue LED (*n* = 12; 27.27%), followed by UV LED (*n* = 10; 22.73%), UV fluorescent (*n* = 9; 20.45%), red LED (*n* = 6; 13.64%), green LED (*n* = 4; 9.09%), and white fluorescent (*n* = 3; 6.82%; Table 2). The Kruskal–Wallis test by ranks showed that *Armigeres* mosquitoes were non-significantly more responsive to UV LED, followed by UV fluorescent, blue LED, red LED, green LED, and white fluorescent, with mean ranks of 116.89, 116.38, 114.56, 105.14, 99.28, and 98.76, respectively (*p* = 0.171; Table 3).

Most individuals with 31.08% (*n* = 46) of *Culex* were collected by using UV fluorescent light, followed by UV LED (*n* = 29; 19.59%), blue LED (*n* = 27; 18.24%), green LED (*n* = 22; 14.86%), white fluorescent (*n* = 17; 11.49%), and red LED (*n* = 7; 4.73%; Table 2). The Kruskal–Wallis test by ranks showed that *Culex* mosquitoes were significantly more responsive to UV florescent, UV LED, and blue LED, with mean ranks of 127.14, 114.57, and 118.94, respectively. Significantly fewer individuals were collected by using green LED, white fluorescent, and red LED, with mean ranks of 106.17, 99.01, and 85.17, respectively (*p* = 0.014; Table 3).

**Figure 2:**
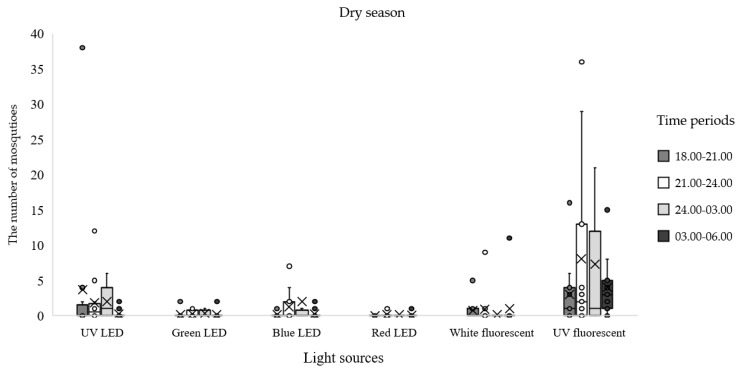
Mean numbers of *Anopheles* mosquitoes caught at different times of night by using different light traps during the dry season, February to May 2020.

**Figure 3:**
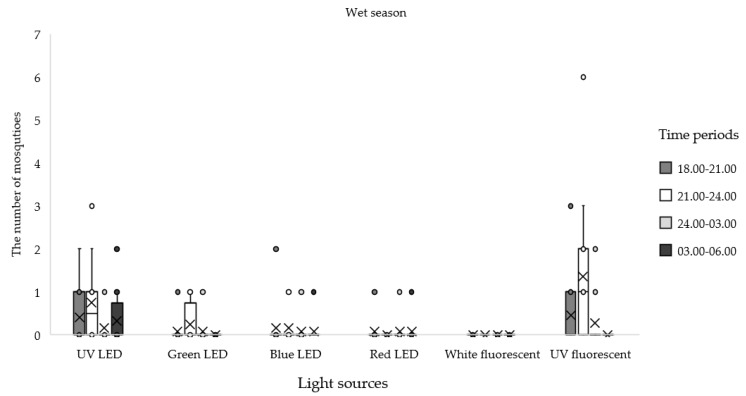
Mean numbers of *Anopheles* mosquitoes caught at different times of night by using different light traps during the wet season, May to October 2020.

**Figure 4:**
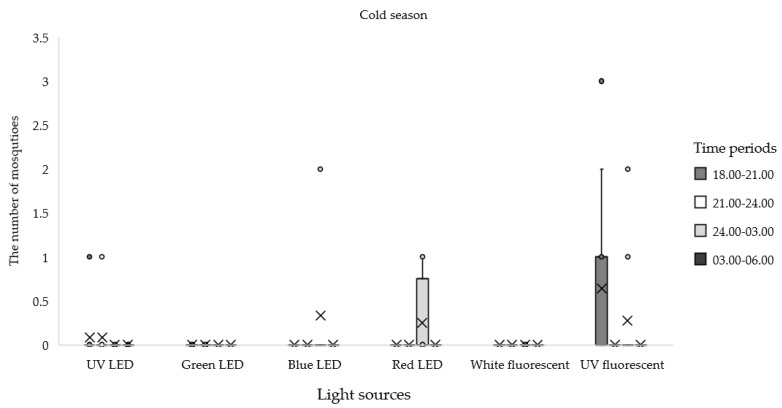
Mean numbers of *Anopheles* mosquitoes caught at different times of night by using different light traps during the cold season, November to February 2020.

When comparing the total number of mosquitoes collected in each season, the greatest proportion of specimens were collected in the dry season (55.76%), followed by the wet (24.63%) and cold (19.70%) seasons. Comparing among nightly collection periods from the entire experiment, the maximum number of mosquitoes was trapped from 21:00 to 24:00 h (31.84%), followed by the time period from 18:00–21:00 h (28.29%), 24:00–03:00 h (24.63%), and 03:00–06:00 h (15.23%). More *Anopheles* mosquitoes were collected in the dry season (*n* = 435; 84.63%) than in the wet (*n* = 60; 11.67%) and cold (*n* = 19; 3.70%) seasons. In the dry season, the trap equipped with UV fluorescent light was the most effective for attracting *Anopheles* mosquitoes; the highest number was collected from 21:00 to 24:00 h, followed by 24:00–03:00 h and 03:00–06:00 h (Table 4; Figure 2). In the wet season, the UV fluorescent light trap was also the most effective for attracting *Anopheles* mosquitoes from 21:00 to 24:00 h (Table 4, Figure 3). In the cold season, the UV florescent trap captured most *Anopheles* mosquitoes from 18:00 to 21:00 h (Table 4; Figure 4).

We used a GLM with negative binomial regression to evaluate the factors (light sources, seasons, and time periods) that influenced the efficacy of light traps to capture *Anopheles* species. Deviance from the goodness-of-fit test at 0.751 and Pearson Chi-square at 1886.706 indicated that the negative binomial regression was suitably obtained (Omnibus test; *p* < 0.001). Light sources, seasons, and collection time periods were all significant predictors that influenced the number of *Anopheles* mosquitoes collected per trap (*p* < 0.05; Table 5). Based on the IRR values, the best efficiency was achieved by using the UV fluorescent light (treated as the standard; IRR = 1), followed by UV LED (IRR = 0.437), blue LED (IRR = 0.202), white fluorescent (IRR = 0.127), green LED (IRR = 0.063), and red LED (IRR = 0.045). The predicted count for mosquito captures in the dry season was 21.649, compared to the cold season as the standard (IRR = 1). In addition, the best trapping time period was 21:00–24:00 h (IRR = 2.54), followed by 24:00–03:00 h (IRR = 2.25) and 06:00–09:00 h (IRR = 1.93), as compared to 03:00–06:00 h as the standard (IRR = 1; Table 5).

## 4. Discussion

Among the four LED wavelength ranges and two fluorescent lights, UV fluorescent was the most effective for mosquito collection, followed by UV LED and blue LED. This is consistent with previous studies that reported the effectiveness of UV fluorescent light for collecting nocturnal mosquitoes [28,40,41]. The UV fluorescent light used in this study had a lower wavelength range (354–468 nm) than those previously evaluated. Breyev et al. [42] reported that more night-biting *Anopheles* mosquitoes, *Anopheles hyrcanus* and *Anopheles maculipennis*, were captured using UV fluorescent light traps (300–400 nm) compared with other light traps of longer wavelengths. This previous study also reported that light traps with spectral beams and 364–400 nm wavelengths increase mosquito attraction [43]. Other previous studies also reported differences in mosquito attraction to LED lights of different wavelengths [18,22,44]. Insect vision responds differently to UV, blue, and green spectra, and responses may vary between species, as well as individuals of the same species inhabiting different areas [21]. Silva et al. [45] reported that light traps with green and blue LEDs attracted more mosquitoes than other LEDs and incandescent lights. In our study, traps with blue LED attracted more mosquitoes than those with green and red LEDs. Although colors of the same brightness are used to evaluate mosquito attraction, physiological light intensities can be affected by differing wavelength absorption in the mosquito eye [46].

In our study, a higher number of *Aedes* species were collected by using the UV fluorescent light trap than the *Culex* species. Similarly, Tchouassi et al. [47] sampled Rift Valley fever vectors, using LED CDC light traps (red, green, and blue) and captured more *Aedes* species than *Culex* species. The lower response of *Culex* species to light traps has not been confirmed but could be attributed to their sensitivity to different wavelengths of light or neurophysiological aspects of their visual systems [48]. Kawada et al. [49] documented that nocturnal host-seeking behavior in nonblood-fed females of *Aedes aegypti* (L.) and *Aedes albopictus* (Skuse) was positively correlated with increasing light intensity. The study used an automatic recording device equipped with a photoelectric sensor and found that the eye of *Ae. aegypti* was highly sensitive to dim light, allowing the species to be active at night [49]. Our results also showed that diurnal *Aedes* mosquitoes were captured during the early evening period. Additionally, Muie et al. [50] reported that the eye of female *Ae. aegypti* has a broad spectral sensitivity, ranging from UV (323 nm) to orange (621 nm) with peaks in the UV (323−345 nm) and green (523 nm) wavelength ranges. Nocturnal *Culex* species’ attraction to light sources differs from other mosquito species [42,51]. In our study, more *Culex* mosquitoes were collected by using the UV fluorescent light trap than other light sources. Our results demonstrate that light source has a significant effect on the number of mosquitoes collected and can be tailored to attract specific genera.

Our light-trap mosquito sampling was conducted from February to December 2020 (dry, wet, and cold seasons). Most *Anopheles* species were collected in the dry season. A previous publication reported that heavy rainfall can flush out larval mosquitoes, resulting in reduced adult densities [52], which is consistent with the lower numbers we observed in the wet season. The best collection times were 21:00–24:00 h and 24:00–03:00 h during the dry season. Harbach et al. [53] reported that mean biting activity of anopheline mosquitoes peaked between 21:00 and 22:00 h Rattanarithikul et al. [54] also reported a prominent peak of *Anopheles* species blood-feeding between 18:00 and 23:00 h. Differences in mosquito behaviors among individuals of the same species have been reported, and are often related to adaptations to human behaviors [55], as well as geographical, climatic, and environmental conditions [7,56,57].

## 5. Conclusions

Trapping *Anopheles* species was found to be most efficient when using light traps fitted with a UV LED light, with the optimal times for collection from 21:00 to 03:00 h in the dry season. We demonstrated that the standard commercial UV fluorescent traps can be replaced with UV LED light traps in sampling *Anopheles* mosquitoes. For future surveillance of adult mosquitoes and incorporation of lights with either the “black hole” styled traps or modifications of other styles of traps, such as CDC, the inclusion of additional chemical lures, such as octanol or lactic acid, may change number of mosquitoes collected, even in the absence of carbon dioxide. This study contributes crucial information for monitoring vector density in regions affected by malaria. A more comprehensive and systematic investigation of mosquito responses to light would be beneficial to the national control program, facilitating more precise vector sampling and monitoring.

## Figures and Tables

**Table 1:** Primers and sequences used for molecular identification of *Anopheles* mosquito species.

Species	Primer Name	Sequence (5′ to 3′)
Universal forward primer	ITS2A	TGT GAA CTG CAG GAC ACA
*Anopheles aconitus*	ACO	ACA GCG TGT ACG TCC AGT
*Anopheles harrisoni*	MIC	GTT CAT TCA GCA ACA TCA GT
*Anopheles varuna*	VAR	TTG ACC ACT TTC GAC GCA
*Anopheles minimus*	MIA	CCC GTG CGA CTT GAC GA
Universal forward primer	5.8F	ATC ACT CGG CTC GTG GAT CG
*Anopheles maculatus*	MAC	GAC GGT CAG TCT GGT AAA GT
*Anopheles pseudowillmoei*	PSEU	GCC CCC GGG TGT CAA ACA G
*Anopheles sawadwongporni*	SAW	ACGGTC CCG CAT CAG GTG C
*Anopheles dravidicus*	PDRAV	GCC TAC TTT GAG CGA GAC CA
Form K	K	TTC ATC GCT CGC CCT TAC AA
Universal forward primer	ITS2A	TGT GAA CTG CAG GAC ACA T
*Anopheles dirus*	D-U	GCG CGG GGC CGA GGT GG
*Anopheles scanloni*	D-AC	CAC AGC GAC TCC ACA CG
*Anopheles cracens*	D-B	CGG GAT ATG GGT CGG CC
*Anopheles baimaii*	D-D	GCG CGG GAC CGT CCG TT
*Anopheles nemophilous*	D-F	AAC GGC GGT CCC CTT TG

**Table 2:** Number and percentage of each adult mosquito species collected by using traps equipped with different light sources.

Mosquitoes	Light Sources						Total No. (%)
	UV LED	Green LED	Blue LED	Red LED	White Fluorescent	UV Fluorescent	
*Aedes* genus	23	31	40	11	19	43	167 (19.13)
*Ae. aegypti*	1	0	1	0	0	0	2 (0.23)
*Ae. albopictus*	15	13	28	0	8	27	91 (10.42)
*Ae. albotaeniata*	0	0	0	1	0	0	1 (0.11)
*Ae. albolateralis*	1	0	1	0	1	0	3 (0.34)
*Ae. chysolineata*	0	2	0	0	1	0	3 (0.34)
*Ae. flaripennis*	1	3	1	2	3	5	15 (1.72)
*Ae. poicilia*	3	0	0	0	2	0	5 (0.57)
*Ae. vexans*	0	0	0	0	0	1	1 (0.11)
*Ae. saxicola*	0	0	1	0	0	1	2 (.023)
*Ae. prominens*	0	0	0	0	1	1	2 (0.23)
*Ae. khazani*	0	0	0	0	1	0	1 (0.11)
*Ae. trilineata*	1	10	6	7	1	2	27 (3.09)
*Ae. mikrokopion*	0	0	1	1	0	0	2 (0.23)
*Ae. lineatopennis*	0	0	0	0	0	1	1 (0.11)
*Ae. pipersalatus*	0	0	0	0	0	1	1 (0.11)
*Aedes* spp. *	1	3	1	0	1	4	10 (1.15)
*Anopheles* genus	115	16	54	11	34	284	514 (58.88)
*An. sawadwongporni*	0	0	1	1	0	6	8 (0.92)
*An. minimus*	1	0	0	1	0	3	5 (0.57)
*An. harrisoni*	105	15	51	8	33	266	478 (54.75)
*An. aconitus*	0	0	1	0	0	0	1 (0.11)
*An. varuna*	3	0	0	0	0	2	5 (0.57)
*An. dirus*	2	0	0	1	0	1	4 (0.46)
*An. barbirostris*	1	0	0	0	0	3	4 (0.46)
*Anopheles* spp. *	3	1	1	0	1	3	9 (1.03)
*Armigeres* genus	10	4	12	6	3	9	44 (5.04)
*Ar.* (*Lei*.) *longipalpis*	5	2	7	5	3	9	31 (3.55)
*Ar. subalbatus*	0	1	2	0	0	0	3 (0.34)
*Ar. achaetae*	1	0	0	0	0	0	1 (0.11)
*Ar. theobaldi*	0	0	0	1	0	0	1 (0.11)
*Ar. leicester*	2	0	0	0	0	0	2 (0.23)
*Armigeres* spp. *	2	1	3	0	0	0	6 (0.69)
*Culex* genus	29	22	27	7	17	46	148 (16.95)
*Cx. brevipalpis*	17	13	11	3	6	23	73 (8.36)
*Cx. tritaeniorhynchus*	7	3	2	3	0	7	22 (2.52)
*Cx. malayi*	1	0	1	0	0	4	6 (0.69)
*Cx. nitropunctatus*	3	1	11	1	8	11	35 (4.01)
*Culex* spp. *	1	5	2	0	3	1	12 (1.37)
Total (%)	177 (20.27)	73 (8.36)	133 (15.23)	35 (4.01)	73 (8.36)	382 (43.76)	873 (100)

* These specimens could not be identified to the species level, due to damage.

**Table 3:** Mean numbers and Kruskal–Wallis mean ranks of four mosquito genera collected during 36 trapping nights, using six different light source traps.

Mosquitoes	Mean ± SD (Mean Rank)					
	UV LED	Green LED	White Fluorescent	UV Fluorescent	Blue LED	Red LED
*Aedes* genus	0.64 ± 0.99 a	0.86 ± 1.91 a	0.52 ± 0.01 a	1.20 ± 1.94 a	1.11 ± 1.95 a	0.31 ± 0.95 a
	(110.89)	(108.58)	(103.39)	(122.92)	(119.03)	(86.19)
*Anopheles* genus	3.19 ± 9.09 a	0.44 ± 1.11 b	0.94 ± 4.16 b	7.89 ± 17.09 a	1.50 ± 4.74 ab	0.31 ± 0.52 b
	(127.82) *	(92.44)	(90.36)	(144.56) *	(103.13)	(92.69)
*Armigeres* genus	0.81 ± 1.39 a	0.61 ± 1.02 a	0.47 ± 0.91 a	1.28 ± 2.11 a	0.75 ± 0.99 a	0.19 ± 0.47 a
	(116.86)	(99.28)	(98.79)	(116.38)	(114.56)	(105.14)
*Culex* genus	0.28 ± 0.51 a	0.11 ± 0.39 ac	0.08 ± 0.28 ac	0.25 ± 0.44 a	0.33 ± 0.76 a	0.17 ± 0.45 bc
	(114.57) *	(106.17)	(99.01)	(127.14) *	(118.94) *	(85.17)

Different letters in each row indicate significant differences among the mean number of mosquitoes collected by each light traps (*p* < 0.05). * Significantly different mean rank, Kruskal–Wallis test.

**Table 4:** Mean number (± SD) of *Anopheles* mosquitoes collected at different times of the night over three seasons, using six different light-source traps.

Seasonal	Light Sources	Total No. (%)	Time Periods			
			Mean ± SD			
			18:00–21:00 h	21:00–24:00 h	24:00–03:00 h	03:00–06:00 h
Dry (12 nights)	UV LED	93 (18.09)	3.67 ± 10.88	1.83 ± 3.51	2.00 ± 2.26	0.25 ± 0.62
	Green LED	11 (2.14)	0.17 ± 0.58	0.25 ± 0.45	0.33 ± 0.65	0.17 ± 0.58
	Blue LED	44 (8.56)	0.17 ± 0.39	1.25 ± 2.22	2.00 ± 6.02	0.25 ± 0.62
	Red LED	5 (0.97)	0.00 ± 0.00	0.17 ± 0.39	0.17 ± 0.39	0.08 ± 0.29
	White Fluorescent	33 (6.42)	0.75 ± 1.42	0.92 ± 2.57	0.17 ± 0.39	0.92 ± 3.18
	UV Fluorescent	249 (48.44)	2.75 ± 4.63	7.67 ± 12.21	6.67 ± 12.60	3.67 ± 4.27
Wet (12 nights)	UV LED	20 (3.89)	0.42 ± 0.67	0.75 ± 0.97	0.17 ± 0.39	0.33 ± 0.65
	Green LED	5 (0.97)	0.08 ± 0.29	0.25 ± 0.45	0.08 ± 0.29	0.00 ± 0.00
	Blue LED	6 (1.17)	0.17 ± 0.58	0.17 ± 0.39	0.08 ± 0.29	0.08 ± 0.29
	Red LED	3 (0.58)	0.08 ± 0.29	0.00 ± 0.00	0.08 ± 0.29	0.08 ± 0.29
	White Fluorescent	1 (0.19)	0.08 ± 0.29	0.00 ± 0.00	0.00 ± 0.00	0.00 ± 0.00
	UV Fluorescent	25 (4.86)	0.58 ± 1.00	1.25 ± 1.82	0.25 ± 0.62	0.00 ± 0.00
Cold (12 nights)	UV LED	2 (0.39)	0.08 ± 0.29	0.08 ± 0.29	0.00 ± 0.00	0.00 ± 0.00
	Green LED	0 (0)	0.00 ± 0.00	0.00 ± 0.00	0.00 ± 0.00	0.00 ± 0.00
	Blue LED	4 (0.78)	0.00 ± 0.00	0.00 ± 0.00	0.33 ± 0.78	0.00 ± 0.00
	Red LED	3 (0.58)	0.00 ± 0.00	0.00 ± 0.00	0.25 ± 0.45	0.00 ± 0.00
	White Fluorescent	0 (0)	0.00 ± 0.00	0.00 ± 0.00	0.00 ± 0.00	0.00 ± 0.00
	UV Fluorescent	10 (1.95)	0.58 ± 1.00	0.00 ± 0.00	0.25 ± 0.62	0.00 ± 0.00

**Table 5:** Incidence rate ratios of factors influencing the efficacy of light traps for capturing *Anopheles* mosquitoes.

Parameter	B	SE	95% Wald Confidence Interval		Hypothesis Test			IRR	95% Wald Confidence Interval for Exp(B)	
			Lower	Upper	Wald Chi-Square	df	Sig		Lower	Upper
(Intercept)	−2.199	0.3026	−2.792	−1.606	52.812	1	0.000	0.111	0.061	0.201
Red LED	−3.111	0.3507	−3.798	−2.423	78.660	1	0.000	0.045	0.022	0.089
Green LED	−2.772	0.3062	−3.372	−2.171	81.914	1	0.000	0.063	0.034	0.114
Blue LED	−1.601	0.2203	−2.033	−1.169	52.849	1	0.000	0.202	0.131	0.311
UV LED	−0.829	0.1928	−1.207	−0.451	18.468	1	0.000	0.437	0.299	0.637
White Fluorescent	−2.065	0.2436	−2.543	−1.588	71.902	1	0.000	0.127	0.079	0.204
UV Fluorescent (Standard)	0 ^2^							1		
Dry Season	3.075	0.2629	2.560	3.590	136.833	1	0.000	21.649	12.932	36.240
Wet Season	1.162	0.2909	0.592	1.732	15.955	1	0.000	3.196	1.807	5.651
Cold Season (standard)	0 ^2^							1		
18:00–21:00 h	0.657	0.2284	0.209	1.104	8.269	1	0.004	1.928	1.233	3.017
21:00–24:00 h	0.933	0.2188	0.504	1.362	18.196	1	0.000	2.543	1.656	3.904
24:00–03:00 h	0.811	0.2228	0.374	1.247	13.244	1	0.000	2.249	1.454	3.481
03:00–06:00 h (Standard)	0 ^2^							1		
(Scale)	1 ^3^									
(Negative binomial)	1 ^3^									

Sig = significance; SE = standard error; df = Degree of Freedom; IRR = incidence rate ratios. ^2^ Set to zero because this parameter is redundant. ^3^ Fixed at the displayed value.

## Data Availability

All relevant data are included in the article.
